# Supplementary material for: Who makes a better university adjustment wingman: Parents or friends?
Source: PLoS One. 2023 Dec 20;18(12):e0294658. doi: 10.1371/journal.pone.0294658 (PMC10732375; doi:10.1371/journal.pone.0294658)
Supplement: S1 File — (DOCX) [file pone.0294658.s002.docx]

Parent and Peer Attachment

Individual Development

(Emerging Adulthood Experiences)

Emerging Adulthood Theory

Attachment Theory

Ecological Transition

(Adjustment in University)

**S1_Fig. The link between attachment theory and emerging adulthood theory (author’s interpretation).**

*
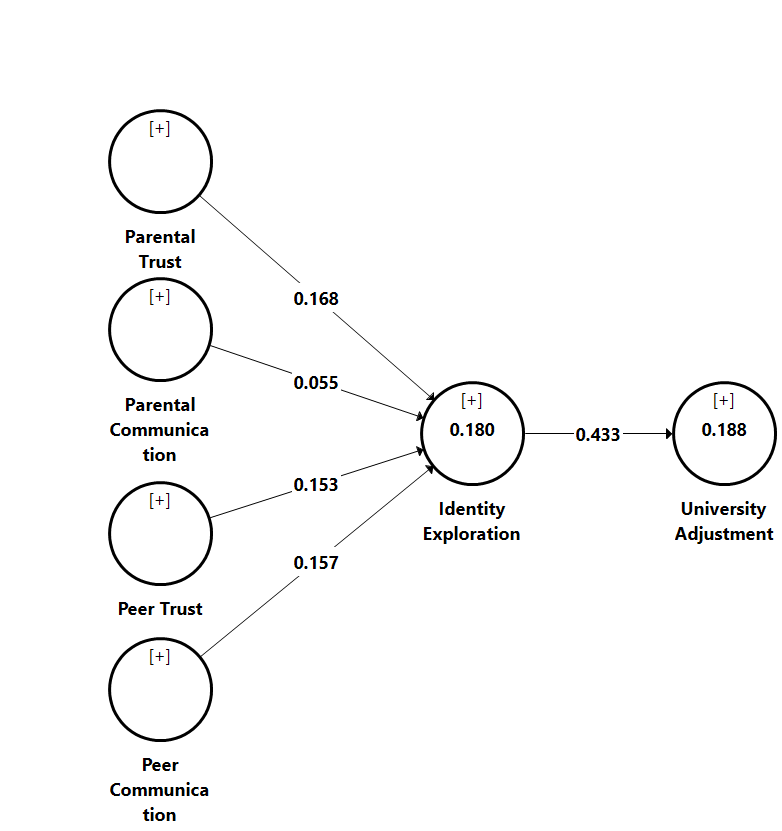
*

**S2_Fig. Results of assessment of structural model.**
