# Supplementary material for: Who makes a better university adjustment wingman: Parents or friends?
Source: PLoS One. 2023 Dec 20;18(12):e0294658. doi: 10.1371/journal.pone.0294658 (PMC10732375; doi:10.1371/journal.pone.0294658)
Supplement: Attachment — Submitted filename: PONE-D-23-18382.pdf [file pone.0294658.s003.pdf]

# PLOS ONE

## Who Makes a Better University Adjustment Wingman: Parents or Friends?

--Manuscript Draft--

| | |
|---|---|
| **Manuscript Number:** | PONE-D-23-18382 |
| **Article Type:** | Research Article |
| **Full Title:** | Who Makes a Better University Adjustment Wingman: Parents or Friends? |
| **Short Title:** | Attachment, Identity Exploration, and University Adjustment |
| **Corresponding Author:** | Walton Wider<br>INTI International University: INTI International University & Colleges<br>Nilai, Malaysia MALAYSIA |
| **Keywords:** | parental attachment; peer attachment; identity exploration; emerging adulthood; university adjustment |
| **Abstract:** | The first year of university is one of the most difficult times in a student's life due to numerous changes that occur. This cross-sectional study explores the concept of parental and peer attachment, which has been researched for its ability to predict students' success in higher education. Yet, less research has investigated the mechanisms underpinning the relationship between attachment and university adjustment among first-year students. Hence, the aim of this study was to examine the impact of parent and peer attachment on first-year university students, and understand how these attachments can facilitate university adjustment through identity exploration. This investigation is underpinned by Bowlby and Ainsworth's attachment theory and Arnett's emerging adulthood theory. Data were collected from 568 first-year students at a public university in Sabah, Malaysia, via adapted questionnaires. Structural equation modelling was employed using SmartPLS Software 3.0 to analyse the data. The study found that identity exploration mediates the relationship between parental trust, peer communication, and university adjustment. The findings of this study provide valuable insights for professionals working with emerging adult clients, especially those in higher education institutions, aiming to enhance the adjustment level among first-year students. |
| **Order of Authors:** | Walton Wider |
| | Jem Cloyd M. Tanucan |
| | Jiaming Lin |
| **Additional Information:** | |
| **Question** | **Response** |
| **Financial Disclosure**<br><br>Enter a financial disclosure statement that describes the sources of funding for the work included in this submission. Review the submission guidelines for detailed requirements. View published research articles from *PLOS ONE* for specific examples.<br><br>This statement is required for submission and **will appear in the published article** if the submission is accepted. Please make sure it is accurate. | The author(s) received no specific funding for this work. |

*Powered by Editorial Manager® and ProduXion Manager® from Aries Systems Corporation*

| | |
|---|---|
| **Unfunded studies**<br>Enter: *The author(s) received no specific funding for this work.*<br><br>**Funded studies**<br>Enter a statement with the following details:<br>• Initials of the authors who received each award<br>• Grant numbers awarded to each author<br>• The full name of each funder<br>• URL of each funder website<br>• Did the sponsors or funders play any role in the study design, data collection and analysis, decision to publish, or preparation of the manuscript?<br>• **NO** - Include this sentence at the end of your statement: *The funders had no role in study design, data collection and analysis, decision to publish, or preparation of the manuscript.*<br>• **YES** - Specify the role(s) played.<br><br>* typeset | |
| **Competing Interests**<br><br>Use the instructions below to enter a competing interest statement for this submission. On behalf of all authors, disclose any competing interests that could be perceived to bias this work—acknowledging all financial support and any other relevant financial or non-financial competing interests.<br><br>This statement is required for submission and **will appear in the published article** if the submission is accepted. Please make sure it is accurate and that any funding sources listed in your Funding Information later in the submission form are also declared in your Financial Disclosure statement.<br><br>View published research articles from *PLOS ONE* for specific examples. | The authors have declared that no competing interests exist. |

| | |
|---|---|
| **NO authors have competing interests**<br><br>Enter: *The authors have declared that no competing interests exist.*<br><br>**Authors with competing interests**<br><br>Enter competing interest details beginning with this statement:<br><br>*I have read the journal's policy and the authors of this manuscript have the following competing interests: [insert competing interests here]*<br><br>* typeset | |
| **Ethics Statement**<br><br>Enter an ethics statement for this submission. This statement is required if the study involved:<br><br>• Human participants<br>• Human specimens or tissue<br>• Vertebrate animals or cephalopods<br>• Vertebrate embryos or tissues<br>• Field research<br><br>Write "N/A" if the submission does not require an ethics statement.<br><br>General guidance is provided below. Consult the submission guidelines for detailed instructions. **Make sure that all information entered here is included in the Methods section of the manuscript.** | Ethical approval was not provided for this study on human participants because the study was conducted according to the guidelines of the Declaration of Helsinki and following academic ethics. The participants provided their written informed consent to participate in this study. |

Powered by Editorial Manager® and ProduXion Manager® from Aries Systems Corporation

| | |
|---|---|
| **Format for specific study types**<br><br>**Human Subject Research (involving human participants and/or tissue)**<br>• Give the name of the institutional review board or ethics committee that approved the study<br>• Include the approval number and/or a statement indicating approval of this research<br>• Indicate the form of consent obtained (written/oral) or the reason that consent was not obtained (e.g. the data were analyzed anonymously)<br><br>**Animal Research (involving vertebrate animals, embryos or tissues)**<br>• Provide the name of the Institutional Animal Care and Use Committee (IACUC) or other relevant ethics board that reviewed the study protocol, and indicate whether they approved this research or granted a formal waiver of ethical approval<br>• Include an approval number if one was obtained<br>• If the study involved *non-human primates*, add *additional details* about animal welfare and steps taken to ameliorate suffering<br>• If anesthesia, euthanasia, or any kind of animal sacrifice is part of the study, include briefly which substances and/or methods were applied<br><br>**Field Research**<br><br>Include the following details if this study involves the collection of plant, animal, or other materials from a natural setting:<br>• Field permit number<br>• Name of the institution or relevant body that granted permission | |
| **Data Availability**<br><br>Authors are required to make all data underlying the findings described fully available, without restriction, and from the time of publication. PLOS allows rare exceptions to address legal and ethical concerns. See the PLOS Data Policy and FAQ for detailed information. | No - some restrictions will apply |

| | |
|---|---|
| A Data Availability Statement describing where the data can be found is required at submission. Your answers to this question constitute the Data Availability Statement and **will be published in the article**, if accepted.<br><br>**Important:** Stating 'data available on request from the author' is not sufficient. If your data are only available upon request, select 'No' for the first question and explain your exceptional situation in the text box.<br><br>Do the authors confirm that all data underlying the findings described in their manuscript are fully available without restriction? | |
| **Describe where the data may be found in full sentences. If you are copying our sample text, replace any instances of XXX with the appropriate details.**<br><br>• If the data are **held or will be held in a public repository**, include URLs, accession numbers or DOIs. If this information will only be available after acceptance, indicate this by ticking the box below. For example: *All XXX files are available from the XXX database (accession number(s) XXX, XXX.).*<br>• If the data are all contained **within the manuscript and/or Supporting Information files**, enter the following: *All relevant data are within the manuscript and its Supporting Information files.*<br>• If neither of these applies but you are able to provide **details of access elsewhere**, with or without limitations, please do so. For example:<br><br>*Data cannot be shared publicly because of [XXX]. Data are available from the XXX Institutional Data Access / Ethics Committee (contact via XXX) for researchers who meet the criteria for access to confidential data.*<br><br>*The data underlying the results presented in the study are available from (include the name of the third party* | Data are available from the Zenodo Institutional Data Access |

| | |
|---|---|
| *and contact information or URL).*<br>• This text is appropriate if the data are owned by a third party and authors do not have permission to share the data.<br><br>* typeset | |
| Additional data availability information: | Tick here if the URLs/accession numbers/DOIs will be available only after acceptance of the manuscript for publication so that we can ensure their inclusion before publication. |

Powered by Editorial Manager® and ProduXion Manager® from Aries Systems Corporation

Manuscript

Click here to access/download;Manuscript;Full Manuscript_14062023.docx

# Who Makes a Better University Adjustment Wingman: Parents or Friends?

Walton Wider[1,*], Jem Cloyd M. Tanucan[2], & Jiaming Lin[1,*]

[1]Faculty of Business and Communications, INTI International University, Nilai, Negeri Sembilan, Malaysia
[2]Cebu Normal University, Cebu, Philippines
*Corresponding Author

## Abstract

The first year of university is one of the most difficult times in a student's life due to numerous changes that occur. This cross-sectional study explores the concept of parental and peer attachment, which has been researched for its ability to predict students' success in higher education. Yet, less research has investigated the mechanisms underpinning the relationship between attachment and university adjustment among first-year students. Hence, the aim of this study was to examine the impact of parent and peer attachment on first-year university students, and understand how these attachments can facilitate university adjustment through identity exploration. This investigation is underpinned by Bowlby and Ainsworth's attachment theory and Arnett's emerging adulthood theory. Data were collected from 568 first-year students at a public university in Sabah, Malaysia, via adapted questionnaires. Structural equation modelling was employed using SmartPLS Software 3.0 to analyse the data. The study found that identity exploration mediates the relationship between parental trust, peer communication, and university adjustment. The findings of this study provide valuable insights for professionals working with emerging adult clients, especially those in higher education institutions, aiming to enhance the adjustment level among first-year students.

*Keywords*: parental attachment; peer attachment; identity exploration; emerging adulthood; university adjustment

## Introduction

Studies on adjustment suggest that it is a diverse and complex phenomenon (Almukhambetova & Hernández-Torrano, 2020). According to previous research, students who are able to adjust at university have better academic results, fewer social problems, and less stress (Malau-Aduli et al., 2021). It is the university's priority to provide adequate services and facilities to encourage adjustment among first-year students (Munday, 2021). The Ministry of Education Malaysia efforts to develop programmes to improve facilities and support programmes are motivated by the fact that many students do not complete their university studies (Ang et al., 2019). Despite the ministry's long-standing efforts, the issue of student adjustment, particularly among first-year students, remains a major concern in Malaysia (Siah et al., 2022). University life has its own set of expectations, including but not limited to students' dedication to the institution, their participation in campus-wide events, their general happiness and success as individuals, and their

academic performance. Adjustment at a university can be seen by looking at personal qualities or behaviour patterns that have the desired value in an environment (Sanagavarapu & Abraham, 2021). A student is said to be adaptable at a university if he is able to socially integrate with the campus residents, is involved in campus activities, meets academic requirements, and is committed to the higher education institution (Zea, Jarama, & Bianchi, 1995). If students are unable to adapt to all of the demands of the university environment, they are likely to drop out of their studies. In order to improve adjustment at university, this study focuses on the factors of parent-peer connection as well as the experience of emerging adulthood from the perspective of identity exploration.

The relationship between attachment and identity has been widely studied and supported by numerous researchers. Ainsworth (1989) and Bowlby (1982) are considered pioneers in the field of attachment theory, which emphasizes the importance of early attachment experiences in shaping an individual's social and emotional development. They proposed that individuals who develop secure attachment styles are more likely to form positive identities and have better social and emotional adjustment throughout their lives. Recent studies have also provided support for the relationship between attachment and identity. De Gregorio et al. (2022) conducted a study exploring the relationship between attachment, identity, and academic performance in college students. They found that individuals with secure attachment styles had a stronger sense of identity and performed better academically. Similarly, Doyle and O'Donnell (2022) found that attachment styles were significantly related to identity formation in adolescents. Kalsner and Pistole (2003) also found that individuals with secure attachment styles had higher levels of identity achievement than those with insecure attachment styles.

Moreover, research has shown that the combination of attachment and identity is important for university adjustment. Kerpelman and Pittman (2018) found that the combination of attachment and identity predicted college adjustment, specifically in terms of academic achievement and social integration. Larose et al. (2019) also found that attachment and identity interacted to predict university adjustment, with individuals who had high levels of both constructs having the best adjustment outcomes. Additional studies have found that attachment is a predictor of identity and, consequently, a high level of adjustment. Lapsley and Edgerton (2002) found that attachment security was positively associated with identity development in late adolescence, and Lapsley et al. (1990) found that individuals with secure attachment styles had better adjustment outcomes in early adulthood. Finally, Schnyders and Lane (2018) and Schnyders et al. (2018) conducted studies exploring the relationship between attachment, identity, and mental health outcomes. They found that individuals with insecure attachment styles and poor identity integration had higher levels of anxiety and depression. In conclusion, the research supports the important relationship between attachment and identity, as well as the combination of both constructs on university adjustment. Attachment is a predictor of identity and, consequently, a high level of adjustment, highlighting the importance of early attachment experiences in shaping individuals' development.

Although some studies have looked at the role of identity exploration as a mediator between parental attachment and university adjustment (see Kaniušonytė and Žukauskienė, 2018;

Lapsley and Edgerton, 2002; Lapsley et al., 1990; Mattanah et al., 2004; Schultheiss and Bluestein, 1994). However, the literature review does not suggest investigating identity as a distinct construct with both positive and negative aspects. As a result, this study suggests that the emerging adulthood experience constructs emerge as a form of identity exploration. The main challenge of emerging adults in university, according to Arnett (2015), is the exploration of identity. There are five emerging adulthood experiences, three of which are positive (identity exploration, self-focus, and a period of possibility) and two of which are negative (instability and feeling "in-between"). As a result, this study adds to the literature by examining the emerging adulthood experience through the lens of identity exploration as a mediator in the relationship between parental attachment, peer attachment, and university adaptation.

**The Importance of Parental and Peer Attachment**

Theoretically, university adjustment is frequently linked to Bowlby and Ainsworth's attachment theory, which examines the parent and peer attachment constructs (Ainsworth, 1989; Bowlby, 1982). Many previous empirical studies have investigated the role of parent and peer attachment on university adjustment (See Mattanah et al, 2011). According to Kenny and Rice (1995), a separation from parents and identity exploration are also active during this sensitive transition period. This results in an unstable period in which students are exposed to stressful and provocative situations (Gillath, Canterberry, & Collins, 2012). In comparison to students who have an insecure attachment to their parents, students who have a secure attachment to their parents have a positive view of academics, are motivated, do good academic work, tend to adapt at university, and have the ability to build a protective emotional, behavioural, and social problem (Darling et al., 2007). A secure relationship with parents can provide students with a sense of acceptance that allows them to try out a new role and make decisions independently while remaining comfortable knowing that their parents support their behaviour. Previous research has shown that attachment shifts from parents to peers as a person grows older (Feldman, 2021). Ainsworth (1989) initially believed that attachment relationships were formed between infants and parents (particularly mothers), but that attachment had shifted to peers as a healthy development among adolescents based on life development. In fact, friendships continue to be important well into adulthood (Schynders & Lane, 2018). Peers are more influential in the adjustment process than parents are during the university years (Grant-Vallone et al., 2003).

**The Importance of Identity Exploration**

Many studies have been discussed in the literature on factors that can influence adjustment at university, and one of them is the exploration of identity (see Arnett, 2016; Azmitia et al., 2013; Berzonsky and Kuk, 2022; Lilgendahl and McLean, 2020). Erikson (1969) proposed that there is a long-term adolescent in a typical industrialised society. Arnett (2000) observed that many young people today have not yet developed a strong commitment to maturity until the age of 25 or more because they are still actively exploring their identity over a long period of time. This stage is known as emerging adulthood. Most studies agree that entering an institutional structure such as a

university increases indicators of well-being among young people (Schulenberg and Zarrett, 2006). The current stage of development at university can foster experiences such as broad opportunities for exploration in a variety of contexts such as cognitive, social, and psychological development, which can influence learning, social activities, and interaction with others (Côté, 2002; Montgomery & Côté, 2003).

**Adjustment as a Life Transitional Process**

For the purpose of this study, Seidman and French's (2004) model of ecological transition and individual development will be utilised to investigate specifically how first-year college students adapt to their new environments. Seidman and French (2004) asserted that the transition process can occur in two ways: either separately and in detail, such as the transition from one state/place to another (eg: entry into the university world), or slowly and gradually, such as the developmental or evolutionary transition from one form or stage to other forms or stages (eg: child and adult development). According to this viewpoint, life transitions can be identified in two ways: ecological transitions and individual developmental transitions.

According to Seidman and French (2004), individual development occurs slowly and gradually, or from one stage to the next (For example, the emerging adulthood period). During the emerging adulthood period, development takes place primarily in five domains: identity exploration, instability, self-focus, feeling "in-between," and full of possibilities (Arnett, 2015). Emerging adulthood period is said to be unstable as a result of changes in planning, such as autonomous relationships with parents and romantic relationships (Arnett, 2015). Adjustment is therefore associated with changes in one's ability to understand others and have new ideas when negotiating relationships with parents (Arnett, 2012). These unique emerging adult experiences develop continuously between the ages of 18 and 29 years (Arnett, 2015) and serve as the foundation for life development in later adulthood. During this time, students are more actively exploring their identities in order to discover who they truly are. Although identity formation begins in adolescence (Erikson, 1963), it is more active and intense during the emerging adulthood period (Arnett, 2000). The period of transitioning also leads to emerging adults becoming more self-focused, i.e., more responsible for themselves and free to make decisions without being influenced by others and not being bound by societal norms in particular (Arnett & Mitra, 2020). The demands and challenges of emerging adulthood are distinct, and adjustment is associated with achieving a level of self-sufficiency (Tanner et al., 2009). To achieve the goal of better understanding themselves, emerging adults are given the freedom to explore their potential and live a changing lifestyle.

An ecological transition occurs when a person's situation in the ecological environment changes due to a change in role, place, or both (Bronfenbrenner, 1979). According to Seidman and French (2004), ecological transitions have two characteristics: normative transitions and non-normative transitions. If a person anticipates an ecological transition, that person is said to have a normative ecological transition. According to Arnett (2000), emerging adulthood normative ecological transitions include work, university, and marriage. These transitions during the

emerging adulthood period demonstrate that changes occur not only in terms of personal development but also in terms of location and residence. When an individual does not anticipate a change in conditions or location, a non-normative ecological transition occurs. The ecological transition is normative in the context of this study because adults develop the expectation that adjustment is required during the transition to university. One of the situations that all emerging adults face is the separation from friends due to a change in residence (Arnett, 2015). As a result, emerging adults must understand how to adapt to social networks and feel a part of a new community (Credé & Niehorster, 2012). Typically, the transition from childhood to adulthood involves not only changes in personal development but also ecological changes that describe an unexpected change in place or role. Individual development and ecological transition, according to Seidman and French (2004), must go hand in hand in the transition of life. If the individual does not experience self-development during the ecological transition, the "developmental mismatch hypothesis", which is based on the concept of person-environment, will occur (Eccles & Midgley, 1989). This concept emphasises the responsibility of the individual to adapt to a specific or appropriate environment. Rationally, emerging adults in an ecological transition, which in the context of this study is a transition in university, require self-development, which is a period of adulthood that develops concurrently. It is necessary to actively explore one's identity during the transition to university in order to improve one's adjustment at university. If one of the transitions fails, it can result in "developmental mismatch", which can lead to a current crisis at the university. This is due to the individual's development status not matching the roles and demands available in the new and foreign university environment.

**Linking Theory of Attachment and Theory of Emerging Adulthood**

The transition to adulthood is said to present ecological and personal development challenges. Emerging adults experience changes in cognitive, biological, emotional, identity, perspective, relationship, achievement, role, responsibility, and other context (Arnett, 2015). From a developmental perspective, the transition to adulthood is unstable. However, some (see Deci and Ryan, 2000; Roberts et al., 2006) see it as a time of excitement and openness. According to Arnett (2015), the vast majority of young adults are choosing to continue their education at a college or university. Higher education institutions are the biggest example of institutions that can offer opportunities and challenges for development that are appropriate for emerging adults (Rarick, 2011). Furthermore, university life is a "social island" distinct from the rest of society, providing a safe haven for emerging adults to explore opportunities in romance, work, and outlook on life (Arnett, 2000; Doyle & O'Donnell, 2022). As a result, the transition to the university world can be seen as an ecological transition that requires adults to adapt and take on new roles. Previous attachment theory research mostly focused on the effects of parent and peer attachment on university adjustment. As such, this study connects the theory of attachment and the theory of emerging adulthood in order to better understand and extend the theoretical framework. The researcher created a conceptual framework for the study by combining attachment theory and emerging adulthood theory. This study's conceptual framework as shown in Figure 1 suggests a

link between parental attachment and peer attachment to individual developmental transitions (emerging adulthood experiences) and then to ecological transitions (university adjustment).

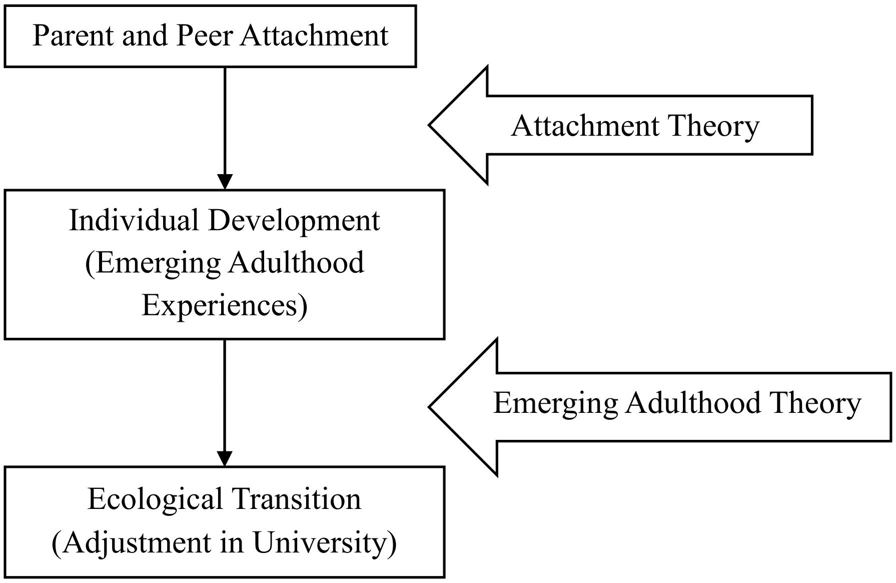


**Figure 1.** The link between attachment theory and emerging adulthood theory

## Method

**Participants**

This cross-sectional study focused on first-year students who were between the ages of 18 and 25 and attended university on a full-time basis. A total of 568 first year students from a public university in Sabah, Malaysia, were involved in this study. G*Power was utilised to determine the minimum sample size required to achieve statistical power (Faul et al., 2009). This study's model includes 4 predictors. Using G*Power with an effect size of 0.15, alpha of 0.05, and power of 0.95, the required minimum sample size was only 129. As a result, our study's sample size of 568 is sufficient, and the results can be confidently reported.

The snowball sampling method was used to recruit participants. The researcher begins by contacting the university lecturers in identifying a few initial participants who meet the desired characteristics, such as being in the aged of 18-29 and full-time public university student. These initial participants were then asked to invite their friends and classmates who also meet the inclusion criteria by sending them the survey link. The process of recruitment then continues with each participant sharing the link or invitation with their network of contacts who also meet the inclusion criteria, and so on. Data was collected over a two-week period in May 2015. A total of 600 questionnaires were collected, however 32 questionnaires were being omitted due to incomplete responses.

Table 1 depicts the demographics of the 568 participants in this study. The majority of respondents are 21 years old (54.9 percent), followed by 20 years old (28.9 percent), 22 years old

(6.7%), 19 to 23 years old (4.0%), 24 years old (0.7%), 25 years old (0.5%), and 18 years old (0.2%). The majority of respondents (66.2%) possess a Malaysian Higher Education Certificate (STPM), followed by the matriculation level (27.1%), the diploma level (6.0%), and the foundation level (0.7%). In terms of gender, women account for 63.7% of respondents, while men account for 36.3%. The majority of respondents are Bumiputera Sabah (48.8%), followed by Malays (23.1%), Chinese (18.1%), others (4.2%), Bumiputera Sarawak (4.0%), and Indians (1.8%).

**Table 1**

*Demographic Profile of Respondents (N=568)*

| Variables | Frequency | % |
|---|---|---|
| Age | | |
| 18 | 1 | 0.2 |
| 19 | 23 | 4.0 |
| 20 | 164 | 28.9 |
| 21 | 312 | 54.9 |
| 22 | 38 | 6.7 |
| 23 | 23 | 4.0 |
| 24 | 4 | 0.7 |
| 25 | 3 | 0.5 |
| Highest level of education | | |
| STPM | 376 | 66.2 |
| Diploma | 34 | 6.0 |
| Matriculation | 154 | 27.1 |
| Foundation | 4 | 0.7 |
| Gender | | |
| Male | 206 | 36.3 |
| Female | 362 | 63.7 |
| Ethnic | | |
| Malay | 131 | 23.1 |
| Chinese | 103 | 18.1 |
| Indian | 10 | 1.8 |
| Bumiputera Sabah | 277 | 48.8 |
| Bumiputera Sarawak | 23 | 4.0 |
| Others | 24 | 4.2 |
| Grade Point Average (GPA) | | |
| 3.50-4.00 | 62 | 10.9 |
| 3.00-3.49 | 258 | 45.4 |
| 2.50-2.99 | 213 | 37.5 |
| 2.4 and below | 35 | 6.1 |

**Instruments**

Section A consisted of questions about sociodemographic profiles, such as gender, age, highest level of education, and ethnic group. The subsequent section encompassed several scales measuring different variables in this study.

*Student Adaptation to College Questionnaire (SACQ)*

The SACQ measurement tool developed by Baker and Siryk (1999) was used to assess university students' adjustment. The SACQ is made up of four sub-scales that assess four aspects of university adjustment (academic, social, personal-emotion, institutional attachment) in first-year students. The SACQ survey tool contains 67 items that use five-point Likert scales (1 = never happens to me, 5 = very often happens to me). Respondents were instructed to respond to the question items by stating how much the statements currently (in the last few days) apply to them. According to the SACQ manual, the institutional adjustment sub-scale contains nine items drawn from the academic (item no. 36) and social (items no. 1, 4, 16, 26, 42, 56, 57, and 65) sub-scales. According to Baker et al. (1985), these nine items correlate to the sub-scale of institutional attachment in their research sample of Clark University students. SmartPLS, on the other hand, cannot analyse items that share sub-scales. As a result, the nine items on the institutional attachment sub-scale were not used in this study. While two items with the numbers 53 and 67 are not used in this study, the researcher who created the SACQ questionnaire never explained why these two items were included in the SACQ questionnaire but not linked to the four sub-scales, resulting in a total of 67 items (Taylor & Pastor, 2005).

*Inventory of Parent and Peers Attachment (IPPA)*

IPPA is comprised of three distinct constructs: trust, communication, and alienation. The revised version of IPPA (Armsden and Greenberg, 1989), containing 50 items, was used for this study. In the first section, the researcher used 25 items, which asked respondents about their attachment to parents or people who act as parents. Meanwhile, the second section contains 25 items in which respondents are asked how they feel about their best friends. Respondents were asked to rate how true the statement was for them right now. Respondents used five-point Likert scales to express their level of agreement (1—almost never, never true, and 5—almost always, always true). In this particular investigation, we focused solely on the trust and communication dimensions of the IPPA instrument.

*Inventory of Dimensions of Emerging Adulthood Experiences (IDEA)*

The IDEA scale is used to assess the identity exploration. Reifman et al. (2007) created this research instrument, which includes 31 question items. This questionnaire examines respondents' relative identification with five distinct dimensions of emerging adulthood: identity exploration, instability, self-focused, feeling "in-between", and possibility. One dimension, the other-focused was omitted from the analysis. This is due to the fact that this dimension does not belong to the dimension of emerging adult experiences. It was designed to contrast with the "self-

focused" subscale. This research tool is scored using four points ranging from 1 (strongly disagree) to 4 (strongly agree). Respondents indicate how much of their current life (as well as the previous few years and the next few years, as they see it) corresponds to each question item.

According to Syed and Mitchell (2013), the dimension of identity exploration in the experience of emerging adulthood consists of four dimensions: instability, possibility, self-focus, and feeling "in-between". According to Arnett (2015), identity exploration is an important part of the emerging adulthood experience because it encompasses all aspects of emerging adulthood experiences. Furthermore, Crocetti et al. (2015) asserted that all dimensions of IDEA are positively and significantly related. As a result of the justification provided, the researcher is able to conceptualise IDEA as identity exploration in this study.

**Pre-test**

After the questionnaire has been formulated, the researcher conducts a pre-test to critically evaluate each item before distributing the questionnaire. It is essential to conduct pre-testing to identify potential issues respondents may have understanding or defining questionnaire questions (Kumar, 2018). A small sample of respondents is used for pre-testing to ensure that the questionnaire questions are clear and easily understood. This is done to reduce problems such as ambiguous and biassed wording, as well as to test the questionnaire's appropriateness in the context study (Sekaran & Bougie, 2016). Convenience sampling was used to select five first-year students to participate in this pre-test. The participants of this pre-test were excluded from the study sample. In addition, two academicians who are experts in the field of developmental psychology have been chosen to provide suggestions and advice for improving the quality of measurement instruments. Each respondent receives a copy of the questionnaire and is instructed to respond, identify any difficulties in understanding the questions and instructions presented on the questionnaire, and evaluate the sentence's relevance to the intended meaning. The researcher identifies and reexamines every comment and suggestion made.

**Data analysis**

This study applies PLS-SEM to gain greater insights into the mediating effect of identity exploration on the relationship between parental trust, parental communication, peer trust, peer communication, and university adjustment. We used PLS-SEM because of its inherent suitability for exploratory studies, which could examine both the measurement and structural models (Sarstedt et al, 2021), using the SmartPLS 3.0 (Ringle et al., 2015) software package. SmartPLS is well-suited for analyzing data from small or moderate sample sizes, which can be common in social science research (Chin, 1998; Risher & Hair, 2017). It uses a partial least squares (PLS) algorithm, which is more robust than traditional maximum likelihood estimation methods for smaller sample sizes (Henseler et al., 2009). SmartPLS is capable of analyzing complex models with multiple latent variables and observed variables (Kamis et al., 2020). It can also handle non-linear relationships and interactions between variables, which can be difficult to analyze using traditional methods (Chin, 1998). Additionally, SmartPLS is able to estimate and test complex

models that include multiple mediators and non-linear relationships between variables. For example, Ringle et al. (2015) demonstrated the use of SmartPLS to estimate a mediation model with multiple mediators and non-linear relationships between variables. For the mediator analysis, the researcher employs the product of the coefficient's method with bootstrapping (Gannon et al., 2020). In addition, demographic factors collected in the study were also tested as possible control variables.

## Results

### Measurement model assessment

Establishing the reliability, convergent validity, and discriminant validity of the measurement model is required. Six reflective constructs were used in this study's framework: parental trust, parental communication, peer trust, peer communication, identity exploration, and university adjustment. A number of measures, including composite reliability (CR) and rho A, were used to assess the measurement model's construct reliability. These criteria must be greater than 0.7. (Hair et al., 2021). Furthermore, to establish convergent validity, the extracted average variance (AVE) should be greater than 0.5. (Ghasemy et al., 2020). Previous research has frequently argued and asked questions about "how many items are appropriate to use to represent a construct in SEM analysis?" According to Hayduk and Littvay (2012), using a few indicators (items) in a study is sufficient, whereas using more than three indicators on a latent variable is less desirable because adding too many indicators provides little benefit in a study, whereas using two items in SEM is reasonable. A small number of items is thought to be the best option because it allows for the development of a parsimonious model with simple explanatory power, increasing the likelihood of model fitness. As a result, it is suggested that the most appropriate items, those with a high loading, be retained. As a result, 58 items from the university adjustment construct, 18 items from identity exploration, 3 items from parental trust, 5 items from parental communication, 2 items from peer trust, and 3 items from peer communication had low loadings and were deleted. As shown in Table 2, the results of the measurement model assessment using the criteria met the required thresholds, indicating that the study model has reliability and convergent validity.

**Table 2**

*Measurement model assessment results*

| Construct | Loadings | rho_A | CR | AVE |
|---|---|---|---|---|
| University Adjustment | | 0.861 | 0.886 | 0.528 |
| ACAD19 | 0.631 | | | |
| ACAD2 | 0.778 | | | |
| ACAD7 | 0.736 | | | |
| INS1 | 0.772 | | | |
| SOC1 | 0.676 | | | |
| SOC4 | 0.694 | | | |
| SOC6 | 0.783 | | | |

| | | | | |
|---|---|---|---|---|
| Identity Exploration | | 0.897 | 0.913 | 0.514 |
| SF5 | 0.658 | | | |
| FIB2 | 0.685 | | | |
| FIB1 | 0.686 | | | |
| SF2 | 0.689 | | | |
| IE5 | 0.700 | | | |
| POSS4 | 0.703 | | | |
| IE7 | 0.717 | | | |
| SF4 | 0.762 | | | |
| IE4 | 0.770 | | | |
| IE6 | 0.788 | | | |
| Parental Trust | | 0.898 | 0.910 | 0.591 |
| PA_TRUST1 | 0.828 | | | |
| PA_TRUST10 | 0.787 | | | |
| PA_TRUST2 | 0.827 | | | |
| PA_TRUST4 | 0.77 | | | |
| PA_TRUST6 | 0.704 | | | |
| PA_TRUST7 | 0.765 | | | |
| PA_TRUST9 | 0.69 | | | |
| Parental Communication | | 0.822 | 0.867 | 0.621 |
| PA_COMM1 | 0.841 | | | |
| PA_COMM3 | 0.824 | | | |
| PA_COMM6 | 0.698 | | | |
| PA_COMM8 | 0.783 | | | |
| Peer Trust | | 0.915 | 0.929 | 0.619 |
| PE_TRUST10 | 0.820 | | | |
| PE_TRUST2 | 0.813 | | | |
| PE_TRUST3 | 0.823 | | | |
| PE_TRUST4 | 0.775 | | | |
| PE_TRUST5 | 0.802 | | | |
| PE_TRUST6 | 0.777 | | | |
| PE_TRUST7 | 0.744 | | | |
| PE_TRUST9 | 0.738 | | | |
| Peer Communication | | 0.855 | 0.885 | 0.607 |
| PE_COMM1 | 0.813 | | | |
| PE_COMM2 | 0.808 | | | |
| PE_COMM3 | 0.816 | | | |
| PE_COMM7 | 0.725 | | | |
| PE_COMM8 | 0.728 | | | |

The researcher used the heterotrait-monotrait (HTMT) ratio to determine the discriminant validity of the constructs in this study (Ghasemy et al., 2020). When the HTMT ratio is less than 0.85 or 0.90, discriminant validity is achieved (Hair et al. 2019). Table 3 shows that the HTMT value for all constructs is less than 0.90, indicating that discriminant validity for this study model has been achieved.

**Table 3**

*Discriminant validity through $HTMT_{0.90}$*

| | | 1 | 2 | 3 | 4 | 5 | 6 |
|---|---|---|---|---|---|---|---|
| 1 | Identity Exploration | | | | | | |
| 2 | Parental Communication | 0.321 | | | | | |
| 3 | Parental Trust | 0.348 | 0.897 | | | | |
| 4 | Peer Communication | 0.393 | 0.403 | 0.382 | | | |
| 5 | Peer Trust | 0.395 | 0.383 | 0.451 | 0.885 | | |
| 6 | University Adjustment | 0.488 | 0.423 | 0.472 | 0.429 | 0.408 | |

**Structural model assessment**

The collinearity between research variables and $R^2$ of the endogenous constructs was investigated when evaluating the structural model and hypothesis testing. T-values and 95% bias-corrected confidence intervals were used to assess the sign and significance of the path coefficient (Hair et al., 2019) and mediation hypothesis (Gannon et al., 2021). Table 4 shows that the VIF values of all constructs were less than 5 (ranged from 1.000 to 2.564), indicating that there was no multi-collinearity problem. $R^2$ values for identify exploration and university adjustment were 0.180 and 0.188, respectively, which are acceptable for behavioural science studies (Hair et al., 2019). Furthermore, the direct effects of parental trust on identity exploration ($\beta$ = 0.168, t = 2.085); peer communication on identity exploration ($\beta$ = 0.157, t = 2.162), and identity exploration on university adjustment ($\beta$ = 0.433, t = 11.542) are significant (Figure 2). However, the direct effect of parental communication on identity exploration ($\beta$ = 0.055, t = 0.835) and peer trust on identity exploration ($\beta$ = 0.153, t = 1.913) are not significant. whereas for mediation analysis, the results support the indirect effects of parental trust ($\beta$ = 0.073, t = 1.937) and peer communication ($\beta$ = 0.068, t = 2.048) on university adjustment through identity exploration (Figure 2). Therefore, this study shows that by engaging in identity exploration, parental trust and peer communication will increase adjustment for university students.

**Control Variable**

In addition to the testing of the proposed links between exogenous and endogenous latent variables as shown in the structural model, one control variable was also examined in this study. GPA added as a control variable in the final model. Control variable is treated as exogenous latent variable similar to other exogenous variables in the model (Kock, 2011). Control variable should be included for the expressed purpose of accounting for known or potential confounding effects

on any construct in the model (Lowry & Gaskin, 2014). GPA is often used as a measure of academic ability or preparedness. Students with higher GPAs are generally perceived to have better academic skills and knowledge, which may make them better equipped to handle the academic demands of university. Therefore, controlling for GPA in studies investigating university adjustment helps to ensure that any observed effects are not simply due to differences in academic ability or preparation.

Secondly, GPA has been found to be associated with various aspects of university adjustment. For example, students with higher GPAs tend to experience less academic stress, have better study habits and time management skills, and are more likely to persist and succeed in their studies. Therefore, controlling for GPA helps to isolate the effects of other variables on university adjustment that are not simply due to differences in academic performance.. In order to test for the effects of control variable in this study GPA was included in the model and linked to university adjustment. The bootstrapping was applied to see the relationship between the control variable and university adjustment. The bootstrapping result indicates GPA has insignificant relationship with university adjustment ($\beta$ = 0.004, t = 0.126), suggesting that identity exploration has no change as the education level change.

**Table 4**

*Results of hypothesis testing*

| Direct/ Indirect Effect | Path Coefficient | t-value | 95% Bias-Corrected Confidence Interval | VIF | Supported |
|---|---|---|---|---|---|
| Parental Trust → Identity Exploration | 0.168 | 2.085 | [0.009, 0.323] | 2.429 | Yes |
| Parental Communication → Identity Exploration | 0.055 | 0.835 | [-0.073, 0.180] | 2.311 | No |
| Peer Trust → Identity Exploration | 0.153 | 1.913 | [-0.016, 0.295] | 2.564 | No |
| Peer Communication → Identity Exploration | 0.157 | 2.162 | [0.022, 0.305] | 2.564 | Yes |
| Identity Exploration → University Adjustment | 0.433 | 11.542 | [0.351, 0.501] | 1.000 | Yes |
| Parental Trust → Identity Exploration → University Adjustment | 0.073 | 1.937 | [0.002, 0.150] | | Yes |
| Parental Communication → Identity Exploration → University Adjustment | 0.024 | 0.831 | [-0.034, 0.078] | | No |
| Peer Trust → Identity Exploration → University Adjustment | 0.066 | 1.871 | [-0.007, 0.131] | | No |
| Peer Communication → Identity Exploration → University Adjustment | 0.068 | 2.048 | [0.009, 0.139] | | Yes |

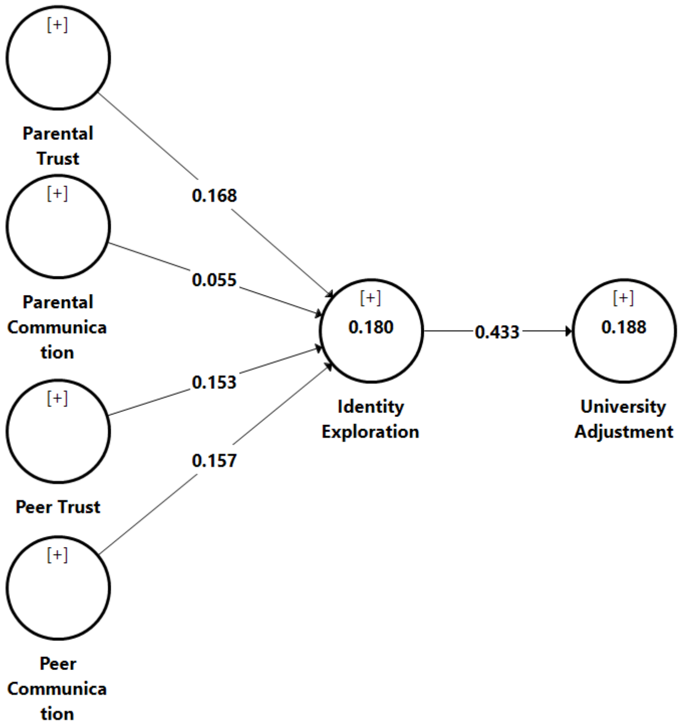


**Figure 2.** *Results of assessment of structural model*

## Discussion

The transition to university brings with it a variety of life demands that can interfere with students' adjustment to university life, particularly among first-year students. As discussed, previous research indicates that the attachment to parents and peers is an important factor that can influence adjustment during the transition to the university. This study introduces the identity exploration as a mediator in an effort to examine how first-year students utilise attachment with parents and peers to achieve identity exploration and further improve university adjustment. The study's findings show that identity exploration acts as a mediator in the relationship between parental trust and university adjustment, but it does not act as a mediator in the relationship between parental communication and university adjustment. In contrast, the findings indicate that identity exploration mediates the relationship between peer communication and university adjustment. However, identity exploration did not act as a mediator in the relationship between peer trust and university adjustment. This study has empirically linked attachment theory and emerging adulthood theory based on the assertions of Seidman and French (2004). According to the findings of this study, parent and peer attachment have similar goals but serve different functions in enhancing identity exploration. Aspects of trust were found to be important in attachment relationships with parents, while communication aspects were found to be important in attachment relationships with peers.

This study also provides strong support for the argument of Kenny and Rice (1995), who assert that starting university is similar to a "strange situation". Emerging adults require a primary

attachment subject (parents), just like infants, to explore new environments. This study also supports Kenny and Donaldson's (1992) argument that university students who have a secure attachment relationship with their parents are better able to adjust to developmental tasks at university. The question also arises as to why the identity exploration does not act as a mediator in the relationship between the communication and adjustment subscale in university. According to Armsden and Greenberg (1989), the behaviour of separation and reunion is evaluated to measure the level of attachment in babies; for adults, the assessment of the level of attachment is seen in terms of the cognitive-affective dimension. This study operationalised the communication sub-scale as "the level and quality of verbal communication with parents". During the transition to college, direct communication with parents diminishes, and parents no longer constantly monitor their children. The decrease in verbal communication between students and parents makes parents unable to respond to or be sensitive to students' emotional states, interfering with the process of student identity development. However, it should be emphasised that even if students have autonomy from their parents, this does not imply that the level of attachment is weak. According to Sullivan and Sullivan (1980), students who live away from their families during the transition period to the university world have a better attachment relationship with their parents than students who live with their parents. The results of this research support this notion, showing a strong association between trust in parents and not for communication with them. According to Armsden and Greenberg (1989), emerging adults view trust as "perceived security" provided by the main attachment subject (parents). In this study, trust is operationally defined as "respect for parents and mutual trust". Students maintain strong bonds by demonstrating mutual respect and trust. Mutual trust between students and parents leads to the exploration of an active and healthy environment, which leads to a good adjustment during the transition to first year at university (Kenny, 1987). This study's findings support the claims of Hiester et al. (2009) and Arnett (2000a) that transition and adjustment at university are important developmental milestones for students because they begin their journey into emerging adulthood at this time. The transition to university life places a high demand on first-year students for autonomy and responsibility. A successful transition to the university environment necessitates students' ability to manage time effectively, create a manageable academic and social schedule, complete academic tasks, consult about a new social life, and respond to new challenges and pressures. This period also includes an extension of the search for self-definition and identity for students in particular. This study also demonstrated that a close relationship between emerging adults and their parents while at university can aid in student development by providing a "safe base" that allows students to explore and build capacity in a life environment outside of the family environment, which in turn can have a positive influence on adjustment to the university in particular.

Studies of emerging adults indicate that during the transition to university, the majority of students will leave home and separate from their parents; this prompts students to seek support from peers to assist them in adjusting to this significant life change (Fraley & Davis, 1997). Attachment theory emphasises that infants have attachment relationships with one primary subject, who is typically the mother, while children begin to develop attachment relationships with other

family members such as siblings or caregivers, and at the adolescent stage and beyond, individuals begin to develop attachment relationships. a diverse group made up of people who are not family members, such as peers. Indeed, attachment theory emphasises the importance of the quality of the attachment relationship, which is not limited to mothers, fathers, and grandparents. In fact, attachment to others is viewed as not causing any major issues or problems in emerging adults. In contrast to peers, the attachment function is more of a "safe haven," in that it helps adults find comfort in the face of threats or fear. Although parent and peer attachment serve different functions, they both serve the same purpose: to be a factor that can assist first-year students in dealing with the transition of identity development and in adjusting to the new university environment. Peers serve as a safe haven, acting as individuals who can provide relief and assistance through the challenges of development and adjustment at university. According to Collins and Repinski (1994), time spent with parents begins to decline in adolescence. Because parents are currently unable to interact face-to-face with students, peers are the closest individuals who can be used as a safe space to express concerns and opinions. According to Nickerson and Nagle (2005), during the growing adulthood period, students tend to turn to peers in desperate situations. Aspects of good communication quality, in particular, can influence the exploration of student identity, where students can share all of their problems and concerns. This study backs up Weimer et al. (2004) claim that good communication with friends indicates the presence of secure relationships with peers, thereby supporting the importance of communication in attachment. Communication with close friends during the period of developing adulthood plays a crucial role in helping adults develop toward identity formation and, at the same time, influences adaptation in the university (Aries & Johnson, 1983).

The study's findings indicate that the aspect of trust in parents has a significant indirect relationship with university adjustment. People dealing with university students such as lecturers, academic advisers, counsellors, and university administrators must understand that parental trust and peer communication must be emphasised in order to encourage students to actively explore the university environment. Starting university requires students to become independent, so they rarely see their families. For this reason, it is crucial that students and parents maintain a level of trust in one another so that college students can confidently explore unfamiliar environments without feeling threatened or uncertain. Additionally, first-year students spend more time with their peers as a result of their independence from their families. Thus, good communication elements must always exist between students and their peers so that students can explore new environments and then make adjustments at university. The university should have a programme that can implement bond formation with parents and peers, particularly in the area of parental trust and peer communications, so that students are more aware of the need to maintain and preserve existing good bonding relationships despite having achieved autonomy from parents. Peers, for example, must function as individuals who can provide a safe space for students to express their problems and concerns while at university. When students discuss problems, their peers must be sensitive and responsive to the opinions expressed. Peers are also encouraged to be more sensitive to things that may upset students' emotions and to be more open in communicating about university

problems. Furthermore, the study's findings suggest that students should be more active in discovering their identity, developing self-awareness, determining their own values and beliefs, and learning to plan for the future. Additionally, students are reminded to have an open mind and to not be afraid to try new things and experiment with different things. Students are also advised to mature gradually, to think optimistically, to be independent, to focus on themselves, to be responsible, and to discover their own abilities. Students should also be aware that the emerging adulthood experience at university can be unstable, with students experiencing confusion, limited feelings, high stress, and anxiety. This is a common side effect of active identity exploration. If the student actively engages in the suggested exploration, he or she will acquire soft skills and become a holistic graduate, that is, a person with good knowledge, morals, behaviour, mindset, and manners as a result of the university learning experience (Abdullah et al., 2006).

## Theoretical Contribution

This study provides empirical evidence that experiences of emerging adulthood (identity exploration) can mediate the relationships between parental attachment, peer attachment, and university adjustment. This study's findings provide a comprehensive conceptual framework based on the argument of Howes and Spieker (2008). This study also establishes an empirical link between Bowlby's attachment theory and Arnett's theory of emerging adulthood, demonstrating how these two theories intersect and are influenced by one another. While attachment theory emphasizes the role of attachment in identity exploration, this study suggests that parent and peer attachment promote identity exploration through distinct mechanisms, such as trust and communication. The study also expands the framework of identity as a distinct construct with both positive and negative aspects, as argued by Syed and Mitchell (2013). Overall, this study contributes to the body of knowledge by examining the mediating effect of identity exploration on the relationships between parental attachment, peer attachment, and university adjustment.

## Practical Contribution

This study has highlighted the significance of parental trust and peer communication to the successful transition of first-year college students. This finding can guide counselors and academicians working with emerging adult students in Malaysian universities, particularly in their efforts to assist first-year students in forming supportive peer relationships. With strong friendships and effective peer communication in college, students will have a safe environment in which to express their problems and concerns, which is essential for their social adjustment. In addition, counselors will be directed to conduct activities that enhance student-parent trust, in light of the knowledge that parental trust is a crucial factor for university adjustment. When students are confident in their parents' trust and support, they can try out a new role at university and make decisions independently while remaining at ease. Finally, with the understanding that identity exploration is a mediator between parental trust and university adjustment, as well as between peer communication and university adjustment, university officials may provide an expansive range of

experiences to students that can help them find answers to questions that surround who they are, including their political and religious views, career choices, and future endeavors.

**Limitations and Future Research**

Despite the fact that the design of this study was chosen based on its suitability to address the study's objectives and key elements, there are still some limitations that the researcher cannot avoid. The first is related to the concept of parental attachment; in this study, respondents are asked to answer questions about the influence of parental attachment without making any distinction between mothers and fathers. According to Bell et al. (1985), the family environment as a whole is more important than the specific relationship with the mother or father in determining the emerging adult's sense of social competence. Among the studies that use a single assessment of parents is the study of Vivona (2000), who justifies this by stating that it is possible unless the respondent has a "very different" relationship with the mother and father, which requires them to assess the mother and father differently. Thus, future research can expand on this study to examine mother and father attachments separately.

Second, the study's limitation related to generalizability is justified by the sampling method and the specific population that was studied. While snowball sampling can be an effective way to recruit participants with specific characteristics, it can also introduce biases into the sample, leading to potential limitations in generalizing the results to other populations. Furthermore, the study was limited to first-year students at one Malaysian institution of higher education. This means that the sample may not be representative of other populations, such as students at different institutions or at different stages of their academic career. Additionally, the cultural context and educational system in Malaysia may differ from other countries, further limiting the generalizability of the results. Therefore, while the findings of the study may be informative and relevant for the specific population and context that was studied, caution should be taken when attempting to generalize the results to other populations or contexts. Nevertheless, the findings of this study as a whole provide new and profound insight and understanding of parental and peer attachment, identity exploration, and university adjustment in the context of first-year students. The researcher believes that assessing this research model from a global perspective by comparing countries and cultural contexts would make this study more interesting. Furthermore, the sample can be drawn by distinguishing the year of study and not limiting the study to first-year students. Previous research has demonstrated that adjustment at university can differ depending on the year of study.

**Conclusions**

The results of this study demonstrate that the attachment theory and the emerging adulthood theory each have their own unique connections during the emerging adulthood period. This study investigates the relationship between parental and peer attachment in the context of the transition to first-year university, as well as the benefits of identity exploration for first-year students. The study discovered that identity exploration serves as a mediator between parental trust

and university adjustment, as well as between peer communication and university adjustment. In conclusion, those who work with university students in particular should be able to identify how factors like parental attachment can influence a successful transition to university life. People who work with emerging adults must also recognise the context of identity exploration, which can influence the relationship between attachment and adjustment during the transition to university life. Furthermore, this study found that relationships with peers are just as important as those with parents, though they can impact adjustment in different ways. Overall, the results of this study lend credence to the notion that first-year students' attachment to both family and friends plays a crucial role in the success of their transition to university life.

## References


Ainsworth, M. S. (1989). Attachments beyond infancy. *American psychologist*, *44*(4), 709–716.

Almukhambetova, A., & Hernández-Torrano, D. (2020). Gifted students' adjustment and underachievement in university: An exploration from the self-determination theory perspective. *Gifted Child Quarterly*, *64*(2), 117-131.

Ang, C. S., Lee, K. F., & Dipolog-Ubanan, G. F. (2019). Determinants of first-year student identity and satisfaction in higher education: A quantitative case study. *SAGE Open*, *9*(2), 2158244019846689.

Aries, E. J., & Johnson, F. L. (1983). Close friendship in adulthood: Conversational content between same-sex friends. *Sex roles*, *9*(12), 1183-1196.

Armsden, G. C., & Greenberg, M. T. (1989). *Inventory of parent and peer attachment (IPPA)*. Seattle: University of Washington.

Arnett, J. J. (2012). New horizons in research on emerging and young adulthood. In *Early adulthood in a family context* (pp. 231-244). Springer, New York, NY.

Arnett, J. J. (2015). *Emerging adulthood: The winding road from the late teens through the twenties*. Oxford University Press.

Arnett, J. J. (2016). College students as emerging adults: The developmental implications of the college context. *Emerging Adulthood*, *4*(3), 219-222.

Arnett, J. J., & Mitra, D. (2020). Are the features of emerging adulthood developmentally distinctive? A comparison of ages 18–60 in the United States. *Emerging Adulthood*, *8*(5), 412-419.

Azmitia, M., Syed, M., & Radmacher, K. (2013). Finding your niche: Identity and emotional support in emerging adults' adjustment to the transition to college. *Journal of research on adolescence*, *23*(4), 744-761.

Baker, R. W., & Siryk, B. (1999). *SACQ: Student adaptation to college questionnaire: Manual*. Western Psychological Services.

Baker, R. W., McNeil, O. V., & Siryk, B. (1985). Expectation and reality in freshman adjustment to college. *Journal of Counseling Psychology*, *32*(1), 94-103.

Bell, N. J., Avery, A. W., Jenkins, D., Feld, J., & Schoenrock, C. J. (1985). Family relationships and social competence during late adolescence. *Journal of youth and adolescence*, *14*(2), 109-119.

Berzonsky, M. D., & Kuk, L. (2022). Identity styles and college adaptation: The mediational roles of commitment, self-agency and self-regulation. *Identity*, *22*(4), 310-325.

Bowlby, J. (1982). Attachment and loss: retrospect and prospect. *American journal of Orthopsychiatry*, *52*(4), 664–678.

Bronfenbrenner, U. (1979). Contexts of child rearing: Problems and prospects. *American psychologist*, *34*(10), 844-850.

Chin, W. W. (1998). Commentary: Issues and opinion on structural equation modeling. *MIS quarterly*, vii-xvi.

Collins, W. A., & Repinski, D. J. (1994). Relationships during adolescence: Continuity and change in interpersonal perspective. In R. Montemayor, G. R. Adams, & T. P. Gullotta (Eds.), *Personal relationships during adolescence* (pp. 7–36). Sage Publications, Inc.

Côté, J. E. (2002). The role of identity capital in the transition to adulthood: The individualization thesis examined. *Journal of youth studies*, *5*(2), 117-134.

Credé, M., & Niehorster, S. (2012). Adjustment to college as measured by the student adaptation to college questionnaire: A quantitative review of its structure and relationships with correlates and consequences. *Educational Psychology Review*, *24*(1), 133-165.

Crocetti, E., Tagliabue, S., Sugimura, K., Nelson, L. J., Takahashi, A., Niwa, T., ... & Jinno, M. (2015). Perceptions of emerging adulthood: A study with Italian and Japanese university students and young workers. *Emerging Adulthood*, *3*(4), 229-243.

Darling, C. A., McWey, L. M., Howard, S. N., & Olmstead, S. B. (2007). College student stress: The influence of interpersonal relationships on sense of coherence. *Stress and Health: Journal of the International Society for the Investigation of Stress*, *23*(4), 215-229.

De Gregorio, A., Vistocco, D., & Dellagiulia, A. (2022). Attachment to Peers and Parents in Italian Catholic Seminarians: Associations with Identity and Well-Being. *Journal of Religion and Health*, 1-22.

Deci, E. L., & Ryan, R. M. (2000). The" what" and" why" of goal pursuits: Human needs and the self-determination of behavior. *Psychological inquiry*, *11*(4), 227-268.

Doyle, C. B., & O'Donnell, G. M. (2022). The theory of emerging adulthood: parents' experience of their child's transition to college. *Higher Education Research & Development*, *41*(2), 330-345.

Eccles, J. S., & Midgley, C. (1989). Stage-environment fit: Developmentally appropriate classrooms for young adolescents. *Research on motivation in education*, *3*(1), 139-186.

Erikson, E. H. (1963). *Youth: Change and challenge*. New York: Basic books.

Feldman, R. (2021). Social behavior as a transdiagnostic marker of resilience. *Annual Review of Clinical Psychology*, *17*, 153-180.

Fraley, R. C., & Davis, K. E. (1997). Attachment formation and transfer in young adults' close friendships and romantic relationships. *Personal relationships*, *4*(2), 131-144.

Friedlander, L. J., Reid, G. J., Shupak, N., & Cribbie, R. (2007). Social support, self-esteem, and stress as predictors of adjustment to university among first-year undergraduates. *Journal of college student development*, *48*(3), 259-274.

Gannon, M., Rasoolimanesh, S. M., & Taheri, B. (2021). Assessing the mediating role of residents' perceptions toward tourism development. *Journal of Travel Research*, *60*(1), 149-171.

Ghasemy, M., Teeroovengadum, V., Becker, J. M., & Ringle, C. M. (2020). This fast car can move faster: A review of PLS-SEM application in higher education research. *Higher education*, *80*(6), 1121-1152.

Gillath, O., Canterberry, M., & Collins, T. J. (2012). A multilevel, multimethod interdisciplinary approach to the understanding of attachment. In O. Gillath, G. Adams, & A. Kunkel (Eds.), *Relationship Science: Integrating Evolutionary, Neuroscience, and Sociocultural Approaches* (pp. 219–240). American Psychological Association.

Grant-Vallone, E., Reid, K., Umali, C., & Pohlert, E. (2003). An analysis of the effects of self-esteem, social support, and participation in student support services on students' adjustment and commitment to college. *Journal of College Student Retention: Research, Theory & Practice*, *5*(3), 255-274.

Hair, J. F., Risher, J. J., Sarstedt, M., & Ringle, C. M. (2019). When to use and how to report the results of PLS-SEM. *European business review*, *31*(1), 2-24.

Hayduk, L. A., & Littvay, L. (2012). Should researchers use single indicators, best indicators, or multiple indicators in structural equation models?. *BMC medical research methodology*, *12*(1), 1-17.

Henseler, J., Ringle, C. M., & Sinkovics, R. R. (2009). The use of partial least squares path modeling in international marketing. In *New challenges to international marketing*. Emerald Group Publishing Limited.

Hiester, M., Nordstrom, A., & Swenson, L. M. (2009). Stability and change in parental attachment and adjustment outcomes during the first semester transition to college life. *Journal of College Student Development*, *50*(5), 521-538.

Kalsner, L., & Pistole, M. C. (2003). College adjustment in a multiethnic sample: Attachment, separation-individuation, and ethnic identity. *Journal of college student development*, *44*(1), 92-109.

Kamis, A., Saibon, R. A., Yunus, F., Rahim, M. B., Herrera, L. M., & Montenegro, P. (2020). The SmartPLS analyzes approach in validity and reliability of graduate marketability instrument. *Social Psychology of Education*, *57*(8), 987-1001

Kaniušonytė, G., & Žukauskienė, R. (2018). Relationships with parents, identity styles, and positive youth development during the transition from adolescence to emerging adulthood. *Emerging adulthood*, *6*(1), 42-52.

Kenny, M. E. (1987). The extent and function of parental attachment among first-year college students. *Journal of youth and Adolescence*, *16*(1), 17-29.

Kenny, M. E., & Donaldson, G. A. (1992). The relationship of parental attachment and psychological separation to the adjustment of first-year college women. *Journal of college student development. 33*(5), 431–438.

Kenny, M. E., & Rice, K. G. (1995). Attachment to parents and adjustment in late adolescent college students: Current status, applications, and future considerations. *The Counseling Psychologist*, *23*(3), 433-456.

Kerpelman, J. L., & Pittman, J. F. (2018). Erikson and the relational context of identity: Strengthening connections with attachment theory. *Identity*, *18*(4), 306-314.

Kock, N. (2011). Using WarpPLS in e-collaboration studies: Mediating effects, control and second order variables, and algorithm choices. *International Journal of e-Collaboration (IJeC)*, *7*(3), 1-13.

Kumar, R. (2018). *Research methodology: A step-by-step guide for beginners*. Sage.

Lapsley, D. K., & Edgerton, J. (2002). Separation□individuation, adult attachment style, and college adjustment. *Journal of Counseling & Development*, *80*(4), 484-492.

Lapsley, D. K., Rice, K. G., & FitzGerald, D. P. (1990). Adolescent attachment, identity, and adjustment to college: Implications for the continuity of adaptation hypothesis. *Journal of Counseling & Development*, *68*(5), 561-565.

Larose, S., Duchesne, S., Litalien, D., Denault, A. S., & Boivin, M. (2019). Adjustment trajectories during the college transition: Types, personal and family antecedents, and academic outcomes. *Research in Higher Education*, *60*(5), 684-710.

Lilgendahl, J. P., & McLean, K. C. (2020). Narrative identity processes and patterns of adjustment across the transition to college: A developmentally contextualized approach. *Journal of Personality and Social Psychology*, *119*(4), 960-977.

Lowry, P. B., & Gaskin, J. (2014). Partial least squares (PLS) structural equation modeling (SEM) for building and testing behavioral causal theory: When to choose it and how to use it. *IEEE transactions on professional communication*, *57*(2), 123-146.

Malau-Aduli, B. S., Adu, M. D., Alele, F., Jones, K., Drovandi, A., Mylrea, M., ... & Jennings, E. (2021). Adjusting to university: Perceptions of first-year health professions students. *PloS one*, *16*(5), e0251634.

Mattanah, J. F., Hancock, G. R., & Brand, B. L. (2004). Parental attachment, separation-individuation, and college student adjustment: A structural equation analysis of mediational effects. *Journal of counseling Psychology*, *51*(2), 213-225.

Mattanah, J. F., Lopez, F. G., & Govern, J. M. (2015). The contributions of parental attachment bonds to college student development and adjustment. *Journal of Counseling Psychology*, *58*(4), 565–596.

Montgomery, M. J., & Côté, J. E. (2003). College as a transition to adulthood. In G. R. Adams & M. D. Berzonsky (Eds.), The Blackwell handbook of adolescence (pp. 149–172). New York, NY: Wiley/Blackwell.

Munday, Z. M. (2021). Adapting to transnational education: students' experiences at an American university in the UAE. *Learning and Teaching in Higher Education: Gulf Perspectives*, *17*(2), 121-135.

Nickerson, A. B., & Nagle, R. J. (2005). Parent and peer attachment in late childhood and early adolescence. *The journal of early adolescence*, *25*(2), 223-249.

Rarick, T. M. (2011). *Importance of perceived adulthood and goal pursuit in emerging adult college students*. Kansas State University.

Reifman, A., Arnett, J. J., & Colwell, M. J. (2007). Emerging adulthood: Theory, assessment and application. *Journal of Youth Development*, *2*(1), 37-48.

Ringle, C., Da Silva, D., & Bido, D. (2015). Structural equation modeling with the SmartPLS. *Brazilian Journal of Marketing, 13*(2), 56-73.

Risher, J., & Hair Jr, J. F. (2017). The robustness of PLS across disciplines. *Academy of Business Journal*, *1*, 47-55.

Roberts, B. W., Walton, K. E., & Viechtbauer, W. (2006). Patterns of mean-level change in personality traits across the life course: a meta-analysis of longitudinal studies. *Psychological bulletin*, *132*(1), 1-25.

Sanagavarapu, P., & Abraham, J. (2021). Validating the relationship between beginning students' transitional challenges, well-being, help-seeking, and their adjustments in an Australian university. *Journal of Further and Higher Education*, *45*(5), 616-628.

Sarstedt, M., Ringle, C. M., & Hair, J. F. (2021). Partial least squares structural equation modeling. In *Handbook of market research* (pp. 587-632). Cham: Springer International Publishing.

Schnyders, C. M., & Lane, J. A. (2018). Gender, parent and peer relationships, and identification with emerging adulthood among college students. *Journal of College Counseling*, *21*(3), 239-251.

Schnyders, C. M., Rainey, S., & McGlothlin, J. (2018). Parent and peer attachment as predictors of emerging adulthood characteristics. *Adultspan Journal*, *17*(2), 71-80.

Schulenberg, J. E., & Zarrett, N. R. (2006). Mental health during emerging adulthood: Continuity and discontinuity in courses, causes, and functions. In J. J. Arnett & J. L. Tanner (Eds.), *Emerging adults in America: Coming of age in the 21st century* (pp. 135–172). American Psychological Association.

Schultheiss, D. E. P., & Blustein, D. L. (1994). Role of adolescent–parent relationships in college student development and adjustment. *Journal of Counseling Psychology*, *41*(2), 248-255.

Sekaran, U., & Bougie, R. (2016). *Research methods for business: A skill building approach*. John Wiley & Sons.

Seidman, E., & French, S. E. (2004). Developmental trajectories and ecological transitions: A two-step procedure to aid in the choice of prevention and promotion interventions. *Development and Psychopathology*, *16*(4), 1141-1159.

Siah, P. C., Ang, S. S., Tan, K. W., Phi, C. N., & Pung, P. W. (2022). Perfectionism and Academic Adjustment among Undergraduates: The Coping Strategy as a Mediator. *Psychological Science and Education*, *27*(5), 57-68.

Sullivan, K., & Sullivan, A. (1980). Adolescent–parent separation. *Developmental Psychology*, *16*(2), 93-99.

Syed, M., & Mitchell, L. L. (2013). Race, ethnicity, and emerging adulthood: Retrospect and prospects. *Emerging adulthood*, *1*(2), 83-95.

Tanner, J. L., Arnett, J. J., & Leis, J. A. (2008). Emerging adulthood: Learning and development during the first stage of adulthood. In *Handbook of research on adult learning and development* (pp. 34-67). Routledge.

Taylor, M. A., & Pastor, D. A. (2007). A confirmatory factor analysis of the Student Adaptation to College Questionnaire. *Educational and psychological measurement*, *67*(6), 1002-1018.

Vivona, J. M. (2000). Parental attachment styles of late adolescents: Qualities of attachment relationships and consequences for adjustment. *Journal of counseling psychology*, *47*(3), 316.

Weimer, B. L., Kerns, K. A., & Oldenburg, C. M. (2004). Adolescents' interactions with a best friend: Associations with attachment style. *Journal of Experimental Child Psychology*, *88*(1), 102-120.

Click here to access/download
**Supporting Information**
STROBE-checklist-v4-combined-PlosMedicine.docx
